# A strategy for pulmonary resection after contralateral diaphragm plication: a surgical case report

**DOI:** 10.1186/s40792-019-0648-z

**Published:** 2019-05-30

**Authors:** Yukiko Matsui, Shigetoshi Yoshida, Takekazu Iwata, Kazuhisa Tanaka, Takayoshi Yamamoto, Kai Nishii, Toshihiko Iizasa

**Affiliations:** 1 Department of Thoracic Surgery, Chiba Cancer Center, 666-2 Nitona-cho, Chuo-ku, Chiba, 260-8717 Japan; 2 Department of Thoracic Surgery, International University of Health and Welfare School of Medicine, Narita, Japan; 3 Department of General Thoracic Surgery, Graduate School of Medicine, Chiba University, Chiba, Japan

**Keywords:** VATS diaphragm plication, CO_2_ insufflation, Multiple lung carcinomas, Pulmonary function

## Abstract

**Abstract:**

**Background:**

Pulmonary carcinoma patients with low pulmonary function cannot be treated surgically because of the high risk of complications. Diaphragmatic eventration is a disease characterized by diaphragmatic paralysis and dyspnea. Here, we report a surgical case of multiple pulmonary carcinomas with contralateral diaphragmatic eventration.

**Case presentation:**

The patient was a 75-year-old woman with multiple metachronous right lung carcinomas complicated by left diaphragmatic eventration. When she was 70 years old, a right upper lobectomy and right S^6^b wedge resection were performed for double lung carcinomas. Five years later, two new lung tumors in her right lower lobe and left diaphragmatic eventration were identified, but resection was thought to be impossible because of her low pulmonary function. We performed video-assisted thoracoscopic surgery (VATS) plication with carbon dioxide (CO_2_) insufflation for the left diaphragmatic eventration, and her pulmonary function improved. Subsequently, we performed a right S^6^ wedge resection and right S^9^ segmentectomy for the double lung tumors with no complications. The tumors were diagnosed as double primary carcinomas.

**Conclusions:**

Our case presented with low pulmonary function and right multiple lung carcinomas with left diaphragmatic eventration. VATS plication for the left diaphragmatic eventration achieved improvement in her pulmonary function, and right pulmonary resection for the lung carcinomas was performed. VATS plication can expand the choice of treatments in such cases.

## Background

Pulmonary function is an important factor to consider for patients with lung diseases when evaluating surgical treatment options. Patients with low pulmonary function cannot be treated surgically because of the high risk of complications.

Diaphragmatic eventration is a disease characterized by diaphragmatic paralysis and dyspnea. Patients with severe symptoms should be considered for surgical treatment.

Here, we report a patient with right multiple lung carcinomas, low pulmonary function, and left diaphragmatic eventration. We performed video-assisted thoracoscopic surgery (VATS) plication using CO_2_ insufflation for the left diaphragmatic eventration. The procedure resulted in the improvement of her respiratory function; subsequently, we performed right pulmonary resection for the lung carcinomas.

## Case presentation

**Fig. 1:**
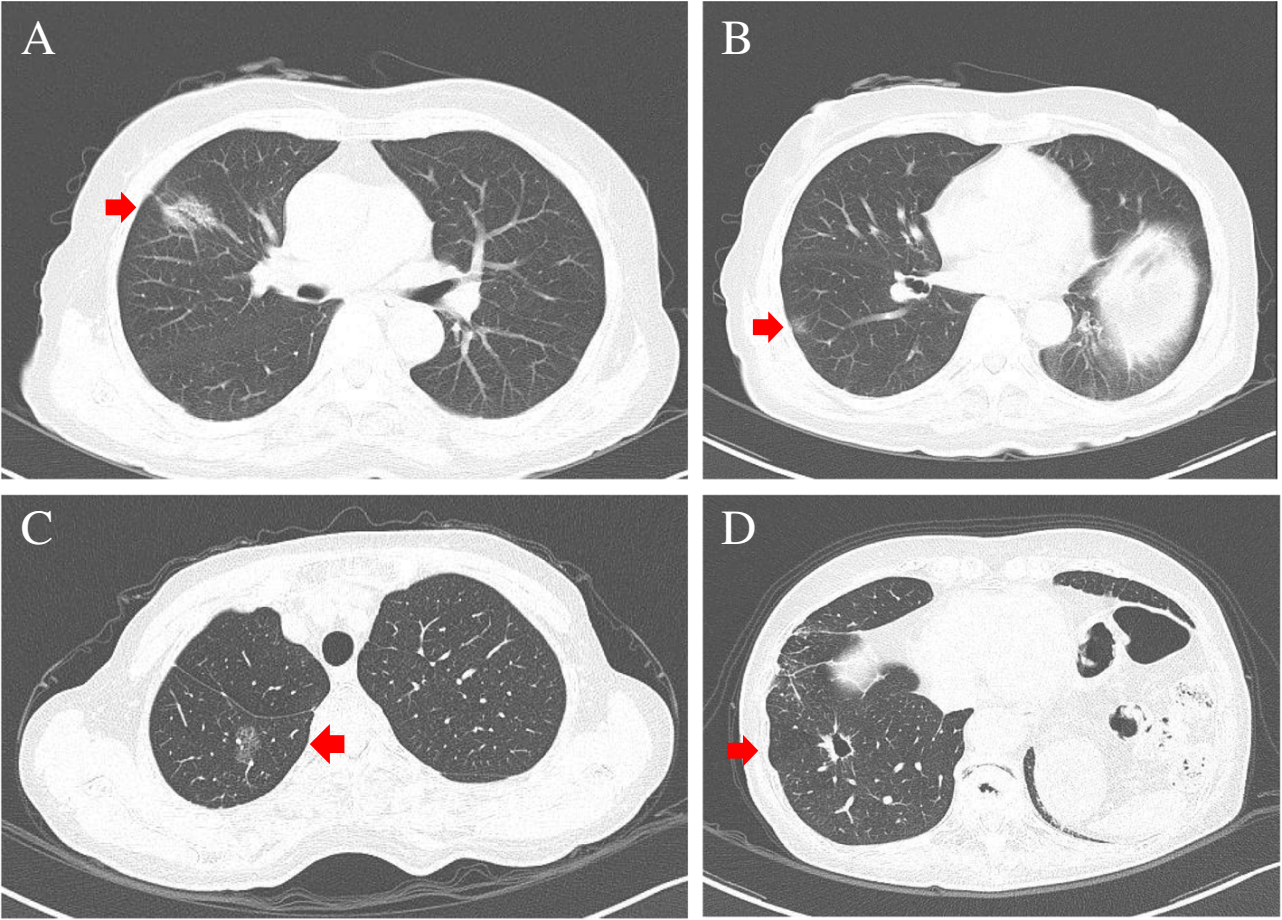
Chest CT showing multiple lung tumors. **a** Right S^2^ tumor. The size was 28 mm. **b** Right S^6^b peripheral tumor. The size was 10 mm. **c** Right S^6^a pure ground-glass nodule. The size was 27 mm. **d** Right S^9^ solid tumor near a bulla. The size was 21 mm

**Fig. 2:**
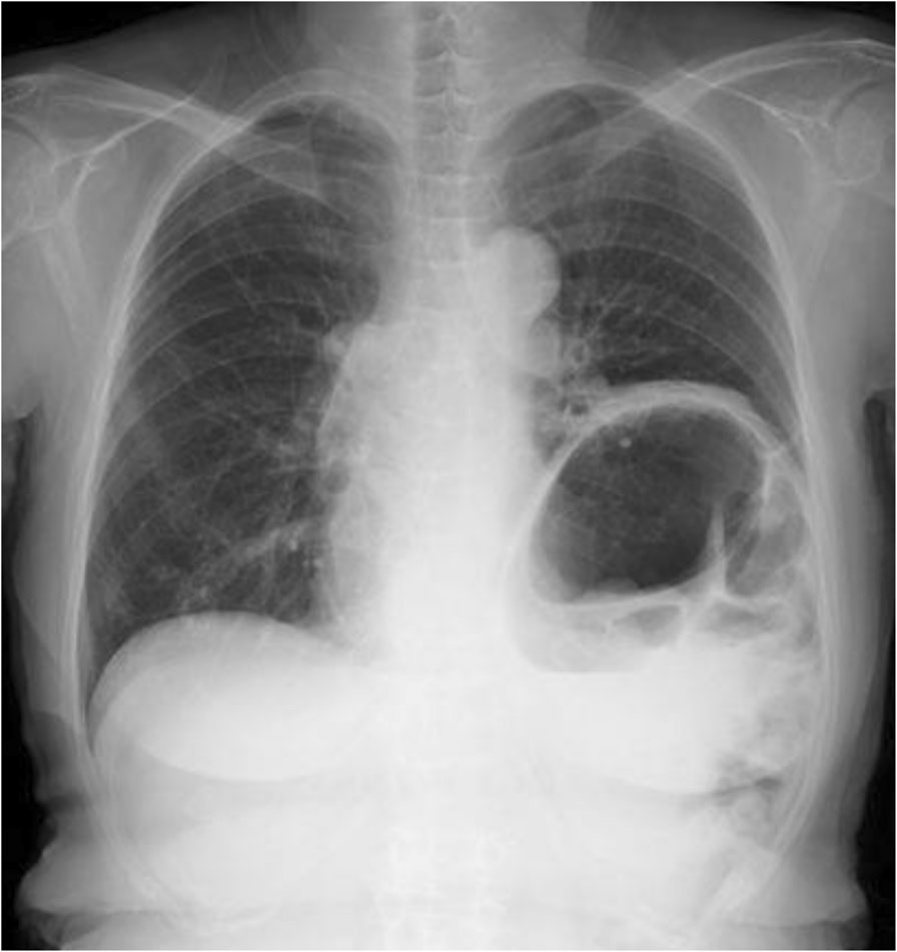
Chest X-ray before diaphragm plication. The left diaphragm was elevated before plication

A 75-year-old woman was admitted in our hospital after a right upper lobectomy and right S^6^b wedge resection for synchronous double lung cancer 5 years previously. The preoperative computed tomography (CT) images showed a right S^2^ tumor and a right S^6^b peripheral tumor (Fig. 1a, b). Metachronous double lung tumors in the right lower lobe (S^6^a and S^9^) and left diaphragmatic eventration were detected with CT and chest X-ray (Figs. 1c, d and 2). The S^6^a tumor was a pure ground-glass nodule with a diameter of 27 mm; there was a distance from the basal segment. The S^9^ tumor diameter was 21 mm, and it was solid and near a bulla in the CT image. The two tumors were suspected to be double primary carcinomas and were located in resectable sites. The patient had mild dyspnea on exertion. The modified Medical Research Council (mMRC) dyspnea score [1] was grade 2. In addition, the result of her pulmonary function test (PFT) was poor; her forced vital capacity (FVC), %FVC, forced expiratory volume in 1 second (FEV_1_), FEV_1_/FVC, and %FEV_1_ were 1.54 L, 70.0%, 0.92 L, 59.7%, and 58.6%, respectively (Table 1). We first concluded that her pulmonary function was too low to undergo pulmonary resection. There was a possibility that her poor pulmonary function was partly due to the left diaphragmatic eventration and that it could be improved with diaphragm plication.

**Table 1:** Change in pulmonary function

	Preoperative	After diaphragm plication	After pulmonary resection
FVC (L)	1.54	2.09	1.49
%FVC (%)	70.0	90.1	69
FEV_1_ (L)	0.92	1.31	1.06
FEV_1_/FVC (%)	59.7	62.7	71.1
%FEV_1_ (%)	58.6	73.2	71.1

*FVC* forced vital capacity, *FEV*_*1*_ forced expiratory volume in one second

**Fig. 3:**
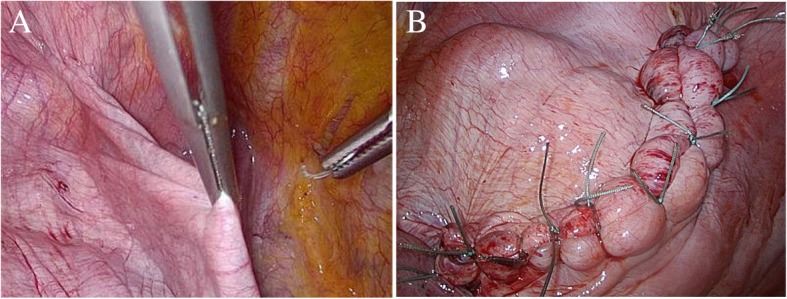
Intraoperative photographs. **a T**he diaphragm was nearly flat using CO_2_ insufflation. **b** After completion of diaphragmatic sutures

We first performed VATS diaphragm plication with CO_2_ insufflation. During the operation, the patient was positioned in a full lateral decubitus position. Four air-locking trocars were placed; the 12-mm trocars were inserted through the fifth intercostal space at the midaxillary line, the seventh intercostal space at the posterior axillary line, and the sixth intercostal space at the anterior axillary line. The 5-mm trocars were placed at the eighth intercostal space at the midaxillary line. CO_2_ gas was insufflated at 8 mmHg pressure through the trocar under thoracoscopic guidance. The diaphragm was sutured from the posterolateral portion to the anteromedial portion using 90-cm-long, #2-0 Ethibond® (Ethicon, USA) threads with interrupted sutures (Fig. 3).

**Fig. 4:**
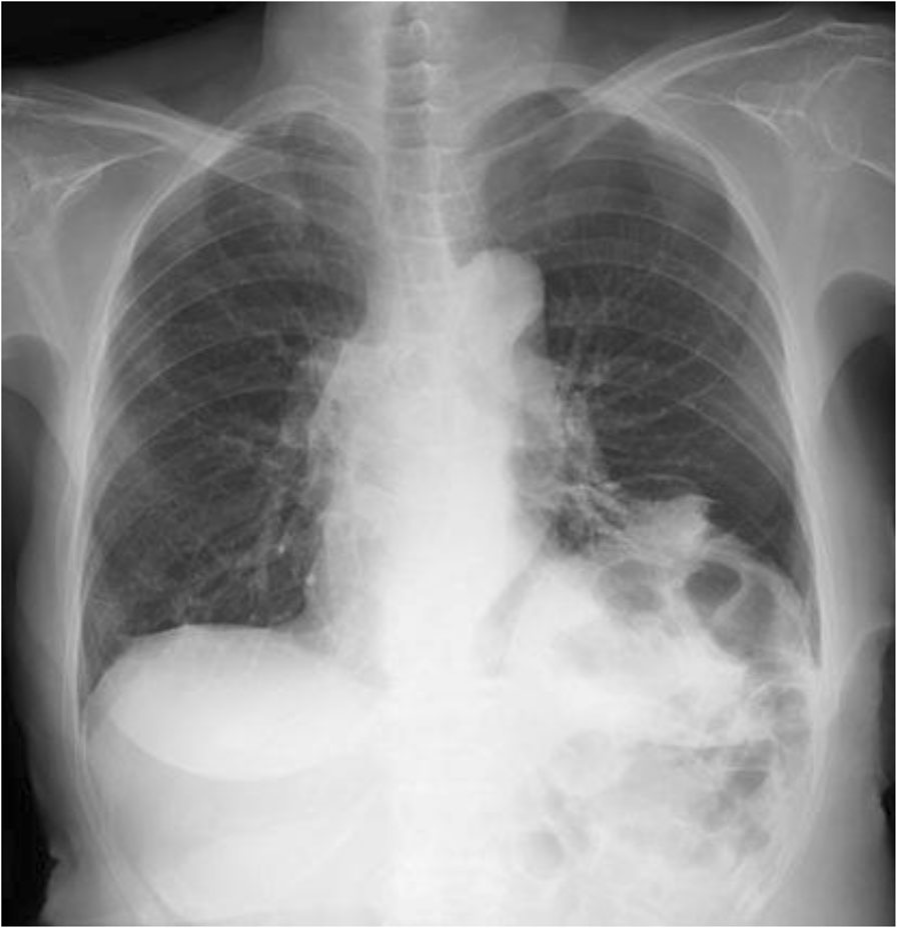
Chest X-ray after diaphragm plication. The improvement in the left lung expansion after plication

Two months after the procedure, she reported a very good improvement in her symptoms, and her PFT showed improvement; her FVC improved to 2.09 L, %FVC to 90.1%, FEV_1_ to 1.31 L, FEV_1_/FVC% to 62.7%, and %FEV_1_ to 73.2% (Table 1). A chest X-ray revealed the expansion of the left lower lung and a flattened left hemidiaphragm (Fig. 4). The result of the arterial blood gas analysis was improved; the preoperative arterial blood oxygen partial pressure (PaO_2_) was 79.3 mmHg and the postoperative PaO_2_ was 93.7 mmHg (Table 2).

**Table 2:** Change in arterial blood gas (room air)

	Preoperative	After diaphragm plication
pH	7.430	7.419
PaO_2_ (mmHg)	79.3	93.7
PaCO_2_ (mmHg)	45.4	42.4
BE (mmol/L)	4.5	2.1
SaO_2_ (%)	96.5	97.6

*PaO*_*2*_ arterial blood oxygen partial pressure, *PaCO*_*2*_ arterial blood carbon dioxide partial pressure, *BE* base excess, *SaO*_*2*_ arterial oxygen saturation

Encouraged by the results, we decided to proceed with lung cancer surgery on the contralateral side and performed a right S^6^ wedge resection and right S^9^ segmentectomy for the double lung tumors. The tumors were diagnosed as double primary carcinomas. The surgical margins of the two tumors were both negative for malignancy. Three months following the last operation, her PFT values were acceptable (Table 1). She was discharged and her respiratory symptoms have not deteriorated to date. The mMRC dyspnea score was grade 1.

## Discussion

This was a case of multiple metachronous carcinomas of the right lung complicated by left diaphragmatic eventration. The decision of which treatment methods to employ in this case was difficult because there were no similar case reports based on our literature search.

The treatment strategies for multiple lung tumors depend on the physical status of the patient. Surgical resections are considered if patients are expected to have sufficient physical functions following resection. An aggressive surgical approach has been reported as a safe and justified method for most patients with multiple primary lung cancers [2]. The selection of surgical methods is important for resection. In our case, the CT image showed that the right S^9^ tumor was 21 mm, solid, and near a bulla. We chose a segmentectomy for the right S^9^ tumor because these characteristics suggested the possibility of high-grade malignancy.

When evaluating the preoperative status of patients, preoperative physiologic assessments are important [3]. In cases where both % predicted postoperative (ppo)-FEV_1_ and %ppo-diffusing capacity of the lungs for carbon monoxide (DLCO) values are less than 60%, the patient is considered to have a high risk for complications following anatomic lung resection [4]. In our case, the initial %FEV_1_ was 58.6%, and we thought the patient had a high risk for pulmonary resection. We nearly abandoned surgical treatment because of her poor pulmonary function; however, we suspected that her poor pulmonary function was caused by the left diaphragmatic eventration and considered performing diaphragm plication to possibly improve her respiratory function. Her pulmonary function improved after diaphragm plication; therefore, this strategy was successful and effective.

Diaphragmatic elevation is a condition caused by phrenic nerve injury or congenital diaphragm eventration. The indications for diaphragm plication are usually dyspnea with low pulmonary function. Diaphragm plication is a well-established surgical procedure that substantially improves dyspnea and objective measures of pulmonary function in patients who are symptomatic from diaphragm paralysis or eventration [5–7]. Diaphragm plication is a safe and effective procedure for adult patients with dyspnea due to unilateral diaphragmatic paralysis [5]. In our case, the patient had mild difficulty breathing. Furthermore, the improvement of her pulmonary function was necessary to safely perform the contralateral lung resection. In symptomatic patients with unilateral diaphragm paralysis, global inspiratory strength is reduced due not only to weakness in the paralyzed hemidiaphragm but also to impairment in the pressure generated by the non-paralyzed hemidiaphragm [8]. In our case, unilateral diaphragm plication might have influenced the contralateral diaphragm movement.

In our case, we needed a minimally invasive surgical technique to treat the diaphragmatic eventration and perform the operation for the contralateral lung carcinomas. We selected VATS with CO_2_ insufflation due to the reduced risk of morbidity compared to that associated with open thoracotomy. The fact that thoracotomy itself is known to reduce diaphragm function transiently [9, 10] and that this deficit recovers more quickly after VATS than after thoracotomy [11] is important when selecting a procedure. Using CO_2_ insufflation for thoracoscopic plication was effective, and VATS with CO_2_ insufflation is becoming common. CO_2_ gas insufflation provided excellent working space and made the stitching easy until the diaphragm was nearly flat [12]. The simplicity of this operation may be safe and is associated with a low risk of morbidity for patients. There is a possibility that the intrathoracic pressure can become too high, but low-pressure insufflation up to 10 mmHg produced no deleterious effects on the patient’s hemodynamic status [13].

## Conclusions

We report a case of low pulmonary function, multiple carcinomas of the right lung and left diaphragmatic eventration. We performed VATS plication for left diaphragmatic eventration and achieved improvement in the patient’s respiratory function. Then, we performed right pulmonary resection for the lung carcinomas. VATS plication may not only lead to an improvement in pulmonary function but also expand the choices for lung disease treatment.

## Data Availability

Not applicable.
